# Characteristics and prognosis of distant metastasis after primary treatment for early‐stage extranodal nasal‐type natural killer/T‐cell lymphoma from the China Lymphoma Collaborative Group database

**DOI:** 10.1002/jha2.613

**Published:** 2022-11-25

**Authors:** Xuan Zheng, Bao‐Lin Qu, Xin Liu, Qiu‐Zi Zhong, Li‐Ting Qian, Yong Yang, Xiao‐Rong Hou, Xue‐Ying Qiao, Hua Wang, Yuan Zhu, Jian‐Zhong Cao, Jun‐Xin Wu, Tao Wu, Su‐Yu Zhu, Mei Shi, Hui‐Lai Zhang, Xi‐Mei Zhang, Hang Su, Yu‐Qin Song, Jun Zhu, Yu‐Jing Zhang, Hui‐Qiang Huang, Ying Wang, Fan Chen, Lin Yin, Xia He, Li‐Ling Zhang, Ye‐Xiong Li, Shu‐Nan Qi

**Affiliations:** ^1^ Department of Radiation Oncology National Cancer Center/National Clinical Research Center for Cancer/Cancer Hospital, Chinese Academy of Medical Sciences and Peking Union Medical College Beijing China; ^2^ Key laboratory of Carcinogenesis and Translational Research (Ministry of Education/Beijing), Department of Radiation Oncology Peking University Cancer Hospital and Institute Beijing China; ^3^ Department of Radiation Oncology The General Hospital of Chinese People's Liberation Army Beijing China; ^4^ Department of Radiation Oncology Beijing Hospital, National Geriatric Medical Center Beijing China; ^5^ Department of Radiation Oncology The Affiliated Provincial Hospital of Anhui Medical University Hefei China; ^6^ Department of Radiation Oncology Fujian Medical University Union Hospital Fuzhou China; ^7^ Department of Radiation Oncology Peking Union Medical College Hospital Chinese Academy of Medical Sciences (CAMS) and Peking Union Medical College (PUMC) Beijing China; ^8^ Department of Radiation Oncology The Fourth Hospital of Hebei Medical University Shijiazhuang China; ^9^ Department of Medical Oncology Second Affiliated Hospital of Nanchang University Nanchang China; ^10^ Department of Radiation Oncology Cancer Hospital of the University of Chinese Academy of Sciences (Zhejiang Cancer Hospital), Zhejiang Key Laboratory of Radiation Oncology Zhejiang China; ^11^ Department of Radiation Oncology Shanxi Cancer Hospital and the Affiliated Cancer Hospital of Shanxi Medical University Taiyuan China; ^12^ Department of Radiation Oncology Fujian Provincial Cancer Hospital Fuzhou China; ^13^ Department of Lymphoma Affiliated Hospital of Guizhou Medical University Guizhou Cancer Hospital Guiyang China; ^14^ Department of Radiation Oncology Hunan Cancer Hospital and the Affiliated Cancer Hospital of Xiangya School of Medicine Changsha China; ^15^ Department of Radiation Oncology Xijing Hospital of Fourth Military Medical University Xi'an China; ^16^ Department of Lymphoma Tianjin Medical University Cancer Institute & Hospital, Key Laboratory of Cancer Prevention and Therapy, National Clinical Research Center for Cancer Tianjin China; ^17^ Department of Radiation Oncology Tianjin Medical University Cancer Institute & Hospital, Key Laboratory of Cancer Prevention and Therapy, National Clinical Research Center for Cancer Tianjin China; ^18^ Department of Lymphoma The Fifth Medical Center of PLA General Hospital Beijing China; ^19^ Key laboratory of Carcinogenesis and Translational Research (Ministry of Education/Beijing), Department of Lymphoma Peking University Cancer Hospital & Institute Beijing China; ^20^ Department of Radiation Oncology Sun Yat‐sen University Cancer Center; State Key Laboratory of Oncology in South China; Collaborative Innovation Center for Cancer Medicine Guangzhou China; ^21^ Department of Medical Oncology Sun Yat‐sen University Cancer Center; State Key Laboratory of Oncology in South China; Collaborative Innovation Center for Cancer Medicine Guangzhou China; ^22^ Department of Radiation Oncology Chongqing University Cancer Hospital and Chongqing Cancer Hospital Chongqing China; ^23^ Department of Radiation Oncology Affiliated Hospital of Qinghai University Qinghai China; ^24^ Department of Radiation Oncology Jiangsu Cancer Hospital and Jiangsu Institute of Cancer Research Nanjing China; ^25^ Cancer Center Union Hospital, Tongji Medical College Huazhong University of Science and Technology Wuhan China

**Keywords:** distant metastasis, NK/T‐cell lymphoma, patterns of metastases, prognosis

## Abstract

This study aimed to investigate the characteristics and prognosis of distant metastasis (DM) after primary treatment for early‐stage extranodal nasal‐type natural killer (NK)/T‐cell lymphoma (ENKTCL). A total of 1619 patients from the China Lymphoma Collaborative Group database were retrospectively reviewed. The cumulative incidence of DM was assessed using Fine and Gray's competing risk analysis. The correlation between DM sites was evaluated using phi coefficients, while DM sites were classified using hierarchical clustering. Regression analysis was used to assess the linear correlation between DM‐free survival (DMFS) and overall survival (OS). The 5‐year cumulative DM rate was 26.2%, with the highest annual hazard rate being in the first year (14.9%). The most frequent DM sites were the skin and soft tissues (SSTs, 32.4%) and distant lymph nodes (LNs, 31.3%). DM sites were categorized into four subgroups of distinct prognosis — distant LN, SST, extracutaneous site, and lymphoma‐associated hemophagocytic lymphohistiocytosis. SST or distant LN, solitary metastasis, and late‐onset DM demonstrated a relatively favorable prognosis. Contemporary chemotherapy significantly decreased DM rates and improved DMFS. Decreased DM rates were further associated with increased OS probabilities. Our findings improve the understanding of the variable clinical behaviors of early‐stage ENKTCL based on four distinct DM sites and thus provide guidance for future therapeutic decisions, metastatic surveillance, and translational trial design.

## 1 INTRODUCTION

Extranodal nasal‐type natural killer (NK)/T‐cell lymphoma (ENKTCL) is rare globally but is relatively common in East Asia and South America [1, 2, 3]. The disease usually originates from the upper aerodigestive tract (UADT), primarily the nasal cavity, and may arise from extranasal‐UADT sites, but rarely from extra‐UADT sites [3, 4, 5]. Most patients (60%–90%) are diagnosed at early stages [2, 3, 4, 5]. Over the past decade, risk‐adapted therapies involving radiotherapy (RT) and non‐anthracycline (ANT)‐based regimens have improved the outcomes of early‐stage ENKTCL, with 5‐year overall survival (OS) rates being 55–90% [6, 7, 8, 9, 10, 11, 12]. RT remains the cornerstone of first‐line treatment [13, 14, 15, 16, 17, 18] and has associated with improved locoregional control, alongside prolonged progression‐free survival (PFS) and OS [13]. Complementing RT with non‐ANT‐based chemotherapy has significantly improved the survival of intermediate‐ and high‐risk early‐stage patients, although not of low‐risk patients.[8, 10, 18] Combined RT and asparaginase (ASP)‐based chemotherapy has also improved the distant metastasis (DM)‐free survival (DMFS) and OS of intermediate‐ and high‐risk early‐stage patients [18].

While early‐stage ENKTCL is curable with modern treatment regimens [19], 10%–35% develop disease progression or relapse after primary treatment,[17, 20–23] depending on the stage and risk profile of the patient.[23, 24] Approximately 15%–30% of patients develop DM,[17, 20–23] while the locoregional failure rate of primary RT has been reported to be approximately 10% [15, 17]. As a progressive disease, DM represents a major clinical issue that can affect patient prognosis. The failure to achieve PFS within 24 months has been associated with a median OS of 5.3 months after disease progression [20]. However, there is currently a lack of large‐scale data on both the failure patterns of primary treatment in early‐stage ENKTCL, as well as the characteristics and outcomes of DM in such patients. Current available studies on DM are limited by their small‐sample monocentric cohorts, and the focus on specific sites such as the central nervous system (CNS), skin, and locoregional areas [25, 26, 27, 28, 29, 30]. With the improvement in OS observed over the past decade [9–11, 18], the clinical outcomes of novel chemotherapy regimens in early‐stage ENKTCL patients have not been fully elucidated.

We thereby performed a large cohort study using the China Lymphoma Collaborative Group (CLCG) database to evaluate the failure patterns of primary treatment for early‐stage ENKTCL, identify the prognostic factors of DM, and assess the effects of contemporary chemotherapy regimens on the DM rates and DMFS of such patients.

## 2 METHODS

### 2.1 Patient eligibility and study population

Patients with newly‐diagnosed early‐stage ENKTCL between 2000 and 2018 from 22 institutions in the CLCG database were retrospectively reviewed. After screening, 1619 patients with complete data were included. This study was approved by the institutional review board, which waived the requirement for informed consent due to the deidentification of patient data. A CONSORT diagram describing the cohort selection is outlined in Figure S1.

### 2.2 Evaluation, primary treatment, and follow‐up

Pre‐treatment evaluation included medical history and physical examination, endoscopy of the UADT, hematology test, computed tomography of the head, neck, chest, abdomen, and pelvis, magnetic resonance imaging of the head and neck, and bone marrow (BM) examination. Positron emission tomography‐computed tomography findings were also recorded. Patients were further risk stratified using the nomogram‐revised risk index (NRI) [24].

Overall, 1126 (69.5%) patients received combined‐modality therapy (CMT), 241 (14.9%) received RT alone, and 252 (15.6%) received chemotherapy alone. Involved‐site RT was administered at a median dose of 53 Gy [6]. Chemotherapy included non‐ANT‐based (*n* = 727, 52.8%), ANT‐based (*n* = 624, 45.3%), and unknown (*n* = 27, 2.0%) regimens. Non‐ANT‐based regimens were further classified into ASP‐based (*n* = 559, 76.9%) and non‐ASP‐based (*n* = 168, 23.1%) regimens.

Follow‐ups were performed every 3 months for the first 2 years, every 6 months until the 5th year, and annually thereafter. The characteristics of DM were assessed by physical examination, imaging, and biopsy if possible. Lymphoma‐associated hemophagocytic lymphohistiocytosis (LAHS) was diagnosed based on previously published diagnostic criteria.[30]

### 2.3 Endpoints and statistical analyses

The primary endpoints included DM rate, OS, and DMFS. DM was defined as the involvement of a distant lymph node (LN) and extranodal site other than the original organ/site or regional LN. OS was defined as the period from the date of initial treatment to the date of death by any cause, while subsequent OS was defined as the period from the date of DM diagnosis to the date of death by any cause. DMFS was defined as the period from the date of initial treatment to the date of DM diagnosis or death.

All categorical data were compared using the chi‐square test. The cumulative incidence of failure was investigated using Fine and Gray's competing risk analysis model. The annual hazard rate of DM was calculated by dividing the annual number of events by the total duration at which the patient was at risk of DM in that year. Hazard rate curves were smoothed using the Epanechnikov kernel function [31]. The correlation between DM sites was evaluated using phi coefficients and was graphically depicted using a heatmap. According to the correlation coefficients calculated, the DM sites were further classified using hierarchical clustering.

Survival data were analyzed using the Kaplan‐Meier method and were compared using the log‐rank test. The inverse probability of treatment weighting (IPTW) approach based on propensity score was used to balance the differences in chemotherapy regimens between the cohorts. Multivariate Cox regression analyses were performed to identify independent prognostic factors for OS. Restricted cubic splines were used to model the non‐linear relationship between time to DM and subsequent OS. Regression analysis was used to assess the linear correlation between DMFS and OS. All statistical analyses were performed using both SPSS version 26.0 (IBM, New York, NY) and the *corrplot, survival, cmprsk*, and *muhaz* package in R version 4.0.2 (http://www.r‐project.org/).

## 3 RESULTS

### 3.1 Baseline clinical characteristics

All baseline clinical characteristics are summarized in Table 1. The male‐to‐female ratio was 2.21:1. Most patients were aged ≤60 years (median age, 43 years; range, 2–85 years), and had good performance status (Eastern Cooperative Oncology Group [ECOG] score 0–1, 95.6%). Elevated lactate dehydrogenase (LDH), primary tumor invasion (PTI), and stage II disease were observed in 26.1%, 58.6%, and 31.9% of the patients, respectively. The majority of primary diseases originated from the nasal cavity (73.5%), followed by extranasal‐UADT sites (23.1%), and extra‐UADT sites (3.4%). According to the early‐stage‐adjusted NRI [24], 23.2%, 35.0%, 28.6%, and 13.3% of the patients were classified into the low‐, intermediate/low‐, intermediate/high‐, and high‐risk groups, respectively.

**TABLE 1:** Clinical characteristics of early‐stage extranodal nasal‐type natural killer/T‐cell lymphoma (ENKTCL) patients stratified by distant metastasis (DM) status

		**DM**	
**Characteristic**	**All patients no. (%)**	**Absence no. (%)**	**Presence no. (%)**	***p***
**Total**	1619	1213	406	
Gender, male	1115 (68.9)	832 (68.6)	283 (69.7)	0.675
Age, >60 years	215 (13.3)	154 (12.7)	61 (15.0)	0.231
B symptoms	666 (41.1)	499 (41.1)	167 (41.1)	0.999
ECOG score, ≥2	71 (4.4)	35 (2.9)	36 (8.9)	<0.001
Stage II	517 (31.9)	354 (29.2)	163 (40.1)	<0.001
PTI	949 (58.6)	677 (55.8)	272 (67.0)	<0.001
Elevated LDH	422 (26.1)	276 (22.8)	146 (36.0)	<0.001
Primary site				<0.001
Nasal	1190 (73.5)	926 (76.3)	264 (65.0)	
Extranasal‐UADT	374 (23.1)	262 (21.6)	112 (27.6)	
Extra‐UADT	55 (3.4)	25 (2.1)	30 (7.4)	
ES‐NRI				<0.001
Low‐risk (0)	375 (23.2)	315 (26.0)	60 (14.8)	
Intermediate/low‐risk (1)	566 (35.0)	447 (36.9)	119 (29.3)	
Intermediate/high‐risk (2)	463 (28.6)	324 (26.7)	139 (34.2)	
High‐risk (≥3)	215 (13.3)	127 (10.5)	88 (21.7)	

Abbreviations: DM, distant metastasis; ECOG, Eastern Cooperative Oncology Group; ENKTCL, extranodal nasal‐type natural killer/T‐cell lymphoma; ES‐NRI, early stage‐adjusted nomogram‐revised risk index.; LDH, lactate dehydrogenase; PTI, primary tumor invasion; UADT, upper‐aerodigestive tract.

With a median follow‐up of 64 months, the 5‐year OS, PFS, and DMFS of the entire cohort were 70.2%, 58.9%, and 64.6%, respectively.

### 3.2 Incidence and annual hazard rate of DM

**FIGURE 1:**
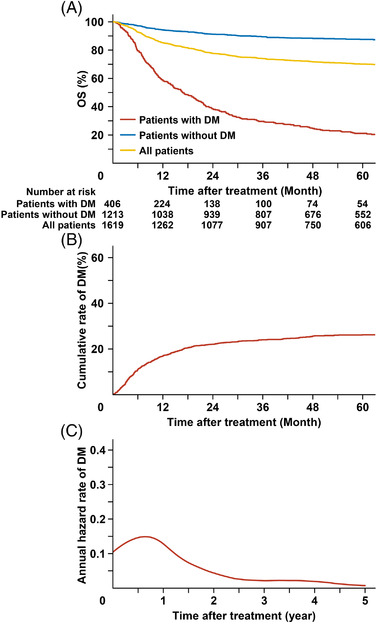
Survival, cumulative rates of DM, and annual hazard rates of DM. (A) The OS of all patients, and those with or without DM. (B) Competing risk analysis of the cumulative rates of DM of the whole cohort. (C) Smoothed hazard plots of DM over time of the whole cohort. DM, distant metastasis; OS, overall survival

Overall, 406 (25.1%) patients developed DM after initial treatment. The 5‐year cumulative rate of DM was 26.2% (Figure 1B). Compared to patients without DM, such patients tended to have more adverse clinical features, such as poor performance status (ECOG score ≥2), Ann Arbor stage II disease, PTI, elevated LDH, and extra‐UADT diseases (Table 1). In multivariable analysis, age > 60, ECOG score ≥2, Ann Arbor stage II disease, PTI, elevated LDH, and extra‐UADT diseases were corelated with worse DMFS (Table S1). The 5‐year OS of patients with and without DM was 21.1% and 87.5%, respectively (*p* < 0.001; Figure 1A).

According to the smoothed hazard plots (Figure 1C), the annual hazard rates of DM was highest in the first year (14.9%), which declined rapidly in the next 2 years. From the third year onwards, the annual hazard rates declined to less than 2.5%.

### 3.3 Distribution and classification of DM

**FIGURE 2:**
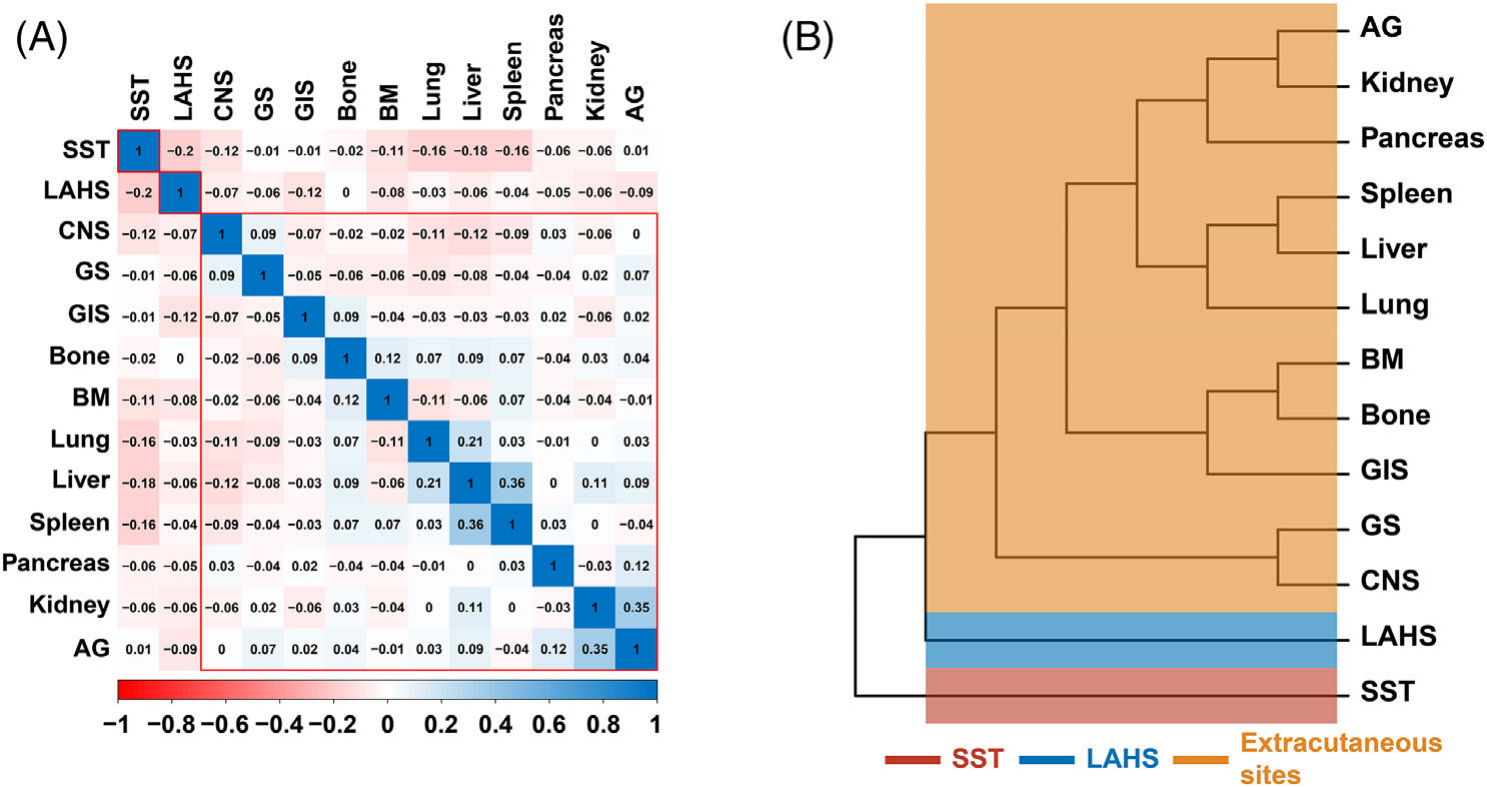
Classification of extranodal DM sites. (A) Heatmap depicting the correlations matrix of all 13 extranodal DM sites. Some extranodal DM sites were negatively correlated (represented in blue), while others were positively correlated (represented in red). (B) Hierarchical clustering of extranodal DM sites based on correlation coefficients. SST, skin and soft tissue; LAHS, lymphoma‐associated hemophagocytic lymphohistiocytosis; CNS, central nervous system; GS, genital system; GIS, gastrointestinal system; BM, bone marrow; AG, adrenal gland

The distribution and classification of DM are shown in Table 2 and Figure 2. Among the 336 patients with available data (Table 2), the most frequent DM sites were the skin and soft tissue (SST, 32.4%) and distant LN (31.3%), followed by the lung (17.3%), liver (14.6%), LAHS (10.7%), and the gastrointestinal system (GIS, 10.4%). The CNS, spleen, urogenital system, adrenal glands, bone, BM, and pancreas were less commonly involved (<10%). The incidence of CNS relapse was low, accounting for 2.0% of all patients, and 8.3% of patients with DM. A total of 302 (89.9%) patients demonstrated extranodal involvement with (*n* = 71) or without (*n* = 231) distant LN metastasis, while 34 (10.1%) patients showed distant LN metastasis only. Visceral involvement of the lung, liver, spleen, CNS, GIS, pancreas, and kidney accounted for 61.6% (*n* = 186) of extranodal metastases.

**TABLE 2:** Distribution of distant metastasis (DM) sites in patients with DM

**DM sites**	**All patients no. (%)**
Total	336 (100)
Distant LN	105 (31.3)
SST	109 (32.4)
CNS	28 (8.3)
Lung	58 (17.3)
Liver	49 (14.6)
Spleen	28 (8.3)
GIS	35 (10.4)
Pancreas	7 (2.1)
AG	23 (6.8)
Kidney	11 (3.3)
GS	23 (6.8)
Bone	18 (5.4)
BM	18 (5.4)
LAHS	36 (10.7)

Abbreviations: AG, adrenal gland; BM, bone marrow; CNS, central nervous system; DM, distant metastasis; GIS, gastrointestinal system; GS, genital system; LAHS, lymphoma‐associated hemophagocytic lymphohistiocytosis; LN, lymph node; SST, skin and soft tissue.

A heatmap based on the correlation coefficients between all extranodal metastatic sites (besides distant LN) was generated (Figure 2A). Patients with SST metastasis or LAHS exhibited negative correlations with other extranodal sites. For instance, SST metastasis was less likely to develop concurrently with liver, spleen, lung or CNS metastasis, or with LAHS. Among the patients with SST metastasis (*n* = 109), 60.0% (*n* = 65) were limited to the skin without extracutaneous dissemination, while 40.0% (*n* = 44) had both cutaneous and extracutaneous dissemination. In contrast, visceral organ dissemination tended to occur simultaneously.

Hierarchical clustering identified three distinct categories of extranodal metastatic sites (Figure 2B). Thus, DM sites were classified into four groups (Table 3) — distant LN (*n* = 34, 10.1%), SST (*n* = 73, 21.7%), extracutaneous site (*n* = 193; 57.4%), and LAHS (*n* = 36, 10.7%).

**TABLE 3:** Classification and definition of distant metastasis (DM) sites

**Category**	**Distant LN**	**SST**	**Other sites**	**LAHS**	**No. (%)**
Distant LN	Present	Absent	Absent	Absent	34 (10.1)
SST	Absent/Present	Present	Absent	Absent	73 (21.7)
Extracutaneous sites	Absent/Present	Absent/Present	Presence	Absent	193 (57.4)
LAHS	Absent/Present	Absent/Present	Absent/Present	Present	36 (10.7)

Abbreviations: DM, distant metastasis; LAHS, lymphoma‐associated hemophagocytic lymphohistiocytosis.; LN, lymph node; SST, skin and soft tissue.

### 3.4 DM Subgroups and their associated outcomes

**FIGURE 3:**
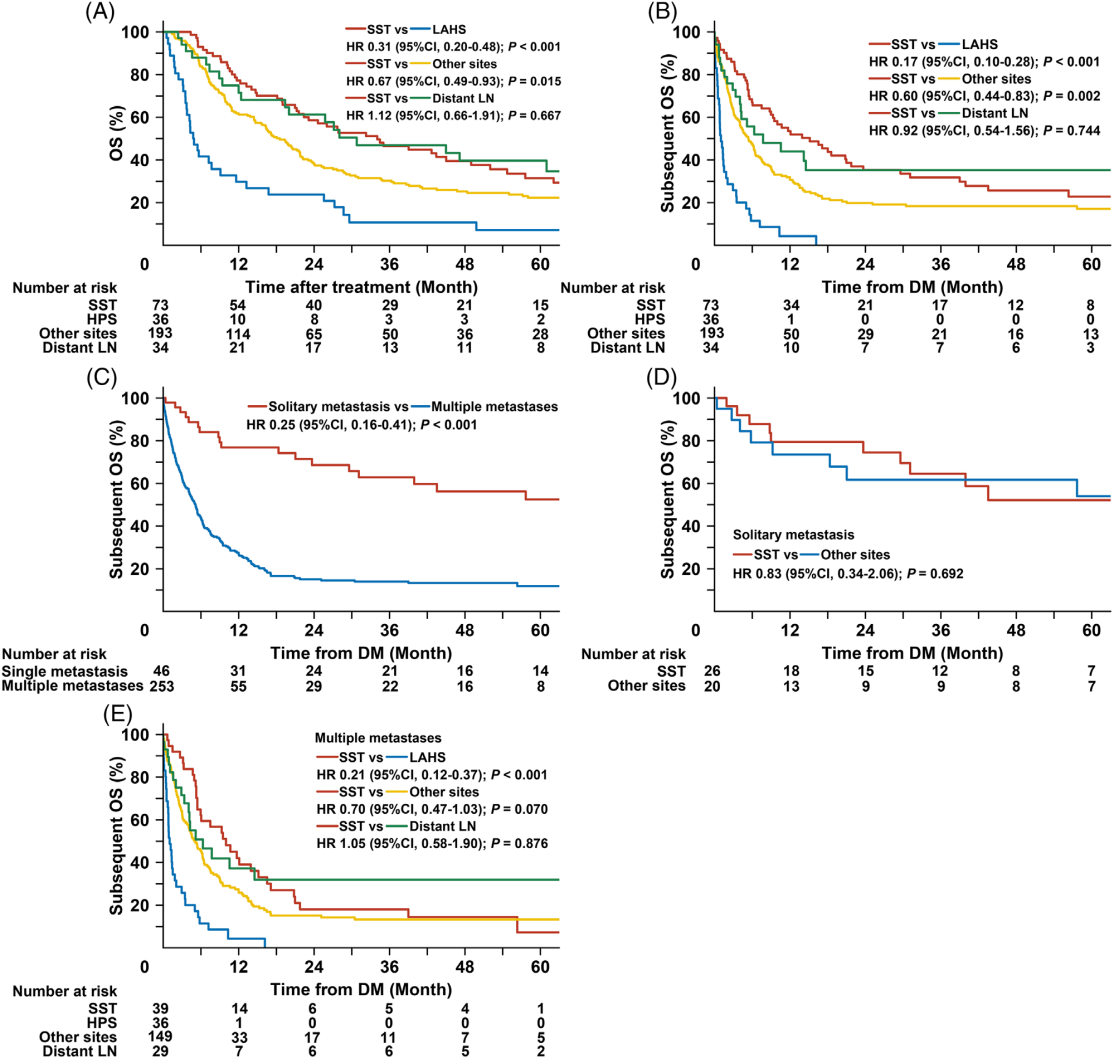
Association of DM site number with the subsequent OS. (A) Comparison of OS between the 4 DM subgroups. (B) Comparison of subsequent OS between the 4 DM subgroups. (C) Comparison of subsequent OS in patients with solitary metastasis and multiple metastases. (D) Comparison of the subsequent OS between solitary metastasis to SST and those of other extracutaneous sites. (E) Comparison of the subsequent OS with multiple metastases based on DM subgroup. DM, distant metastasis; OS, overall survival; HR, hazard ratio; CI, confidence interval; SST, skin and soft tissue; LAHS, lymphoma‐associated hemophagocytic lymphohistiocytosis; LN, lymph node

The four DM subgroups showed different prognoses. The median survival of all patients with DM was 4.34 months. The 5‐year OS was comparable between the SST and distant LN groups (31.5% vs. 39.7%; Hazard ratio (HR) 1.12; 95% confidence interval (CI) 0.66–1.91; *p* = 0.667; Figure 3A). However, the SST group showed higher 5‐year OS compared with the extracutaneous site group (31.5% vs. 22.3%; HR 0.67; 95% CI 0.49–0.93; *p* = 0.015) and the LAHS group (31.5% vs. 7.1% [median, 4.8 months]; HR 0.31; 95% CI 0.20–0.48; *p* < 0.001) groups. Based on multivariable analysis (Table 4), all DM subgroups were found as independent prognostic factors for OS.

**TABLE 4:** Multivariable analysis of overall survival (OS) for early‐stage patients with distant metastasis (DM)

	**OS**	
**Variables**	**HR (95% CI)**	***p***
Gender (female vs. male)	0.84 (0.64–1.11)	0.215
Age (>60 vs. ≤60)	1.11 (0.76–1.63)	0.599
B symptoms (yes vs. no)	1.00 (0.77–1.31)	0.976
ECOG score (≥2 vs. 0–‐1)	0.76 (0.47–1.24)	0.267
Ann Arbor stage (II vs. I)	1.48 (1.12–1.96)	0.006
Elevated LDH (yes vs. no)	1.20 (0.92–1.56)	0.183
PTI (yes vs. no)	1.37 (1.03–1.84)	0.034
Primary site		0.002
Extranasal‐UADT vs nasal	0.70 (0.51–0.96)	0.027
Extra‐UADT vs nasal	0.39 (0.21–0.72)	0.003
DM groups		<0.001
SST vs LAHS	0.28 (0.18–0.44)	<0.001
SST vs extracutaneous sites	0.63 (0.45–0.88)	0.006
SST vs distant LN	1.07 (0.63–1.84)	0.796

Abbreviations: CI, confidence interval; DM, distant metastasis; ECOG, Eastern Cooperative Oncology Group; HR, hazard ratio; LAHS, lymphoma‐associated hemophagocytic lymphohistiocytosis; LDH, lactate dehydrogenase; LN, lymph node.; OS, overall survival; PTI, primary tumor invasion; SST, skin and soft tissue; UADT, upper‐aerodigestive tract.

Similarly, the SST group demonstrated higher subsequent 3‐year OS compared with the extracutaneous site group (31.8% vs. 18.4%; HR 0.60; 95% CI 0.44–0.83; *p* = 0.002; Figure 3B) and the LAHS group (31.8% vs. 0% [median, 1.0 months]; HR 0.17; 95% CI 0.10–0.28; *p* < 0.001). No significant difference in subsequent OS was observed between the SST and distant LN groups (31.8% vs. 35.2%; HR 0.92; 95% CI 0.54–1.56; *p* = 0.744). These results indicated that the proposed DM classification predicted OS independently.

### 3.5 Association of the number of DM sites with subsequent OS

We evaluated the association between the number of DM sites and subsequent OS. Of the 299 patients with available data, 253 (84.6%) and 46 (15.4%) developed multiple and solitary metastases, respectively. Patients with solitary metastasis showed significantly higher 3‐year subsequent OS than those with multiple metastases (62.9% vs. 14.0%; HR 0.25, 95% CI 0.16–0.41; *p* < 0.001; Figure 3C).

The most common site of solitary metastasis was the SST (*n* = 26, 56.5%), followed by the genital system (*n* = 7, 15.2%). Other involved sites included the lung (*n* = 3), GIS (*n* = 2), adrenal gland (n = 2), kidney (*n* = 2), liver (*n* = 2), CNS (*n* = 1), and distant LN (*n* = 1). Comparable 3‐year subsequent OS was seen between solitary metastasis of the SST and extracutaneous sites (64.5% vs. 61.7%; HR 0.83, 95% CI 0.34–2.06; *p* = 0.692; Figure 3D).

**FIGURE 4:**
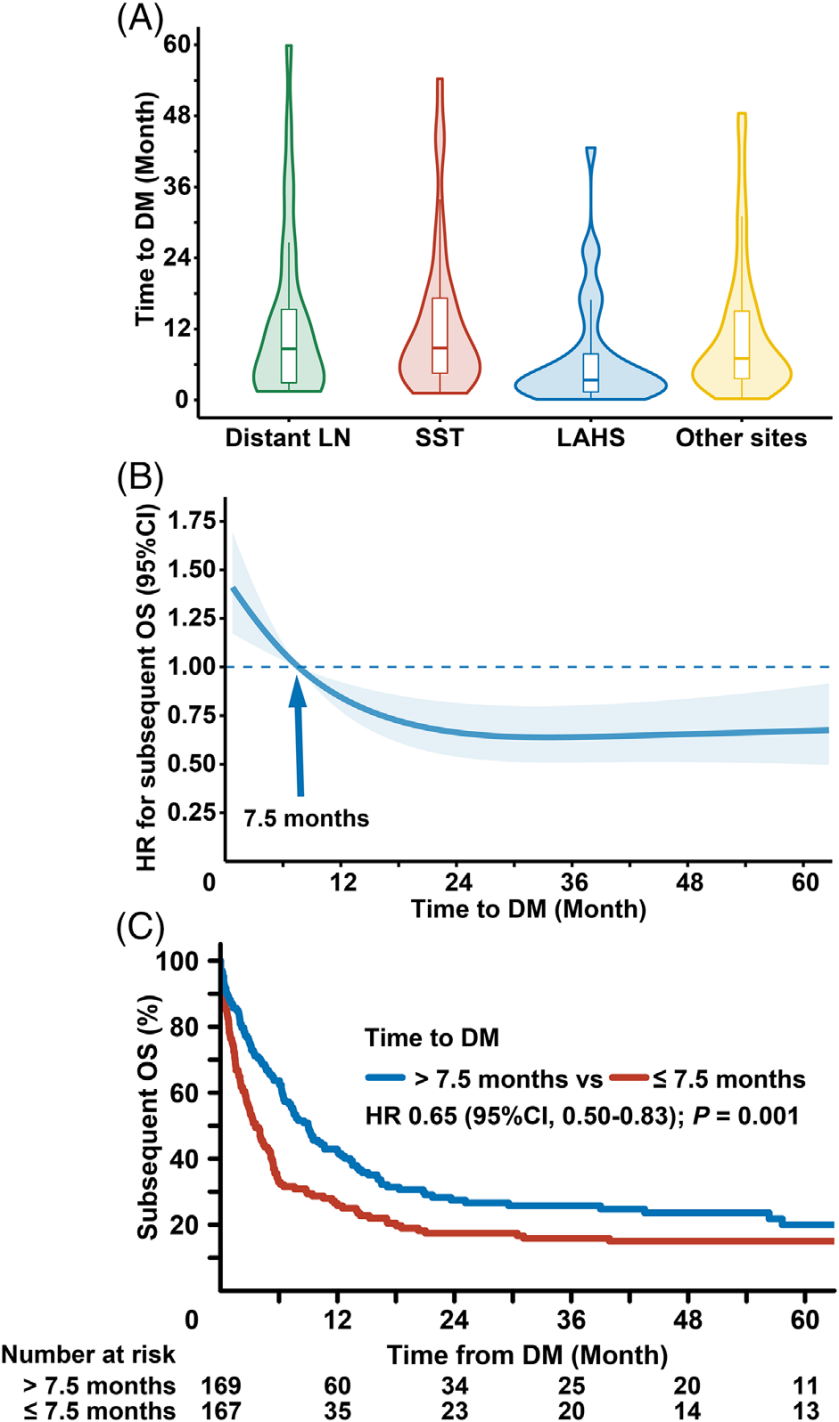
Association of time to DM with subsequent OS. (A) Violin plot depicting the time to DM of each DM subgroup. (B) Restricted cubic splines for the association between time to DM and estimated HRs for the subsequent OS. Solid lines represent the HRs, while shaded areas represent the 95% CIs. Knots were placed at the 5th, 50th, and 95th percentiles of time to DM. A reference point was set at 7.5 months (median time to DM). (C) Comparison of subsequent OS between patients with early‐onset DM and late‐onset DM using 7.5 months as the cutoff. DM, distant metastasis; OS, overall survival; SST, skin and soft tissue; LAHS, lymphoma‐associated hemophagocytic lymphohistiocytosis; LN, lymph node; HR, hazard ratio; CI, confidence interval

For patients with multiple metastases, the subsequent 3‐year OS of the SST group was significantly higher than that of the LAHS group (18% vs. 0% [median, 1.0 months]; HR 0.21; 95% CI 0.12–0.37; *p* < 0.001; Figure 4E), but was similar to that of the extracutaneous sites group (18.0% vs. 13.3%; HR 0.70; 95% CI 0.47–1.03; *p* = 0.070) and the distant LN group (18.0% vs. 31.9%; HR 1.05; 95% CI 0.58–1.90; *p* = 0.876). These results indicated that prognosis in patients with DM associated with the number of DM sites.

### 3.6 Association of time to DM with subsequent OS

The time to DM of each patient in the four DM subgroups is presented in a violin plot (Figure 4A). The median time to DM varied between subgroups, from 10.4 months for the distant LN group, 9.0 months for the SST group, 7.7 months for the extracutaneous site group, to 3.7 months for the LAHS group. The relationship between time to DM and subsequent OS is illustrated using a restricted cubic spline (Figure 4B, *P* for nonlinearity = 0.001). The risk of death decreased rapidly within the first 7.5 months, and plateaued after 24 months. Using 7.5 months as a cutoff point, patients with late‐onset DM showed better 3‐year subsequent OS compared to those with early‐onset DM (25.8% vs. 15.8%; HR 0.65; 95% CI 0.50–0.83; *p* = 0.001; Figure 4C). These results indicated that early‐onset DM associated with poorer OS.

### 3.7 Association of contemporary chemotherapy regimens with DM rates and DMFS

**FIGURE 5:**
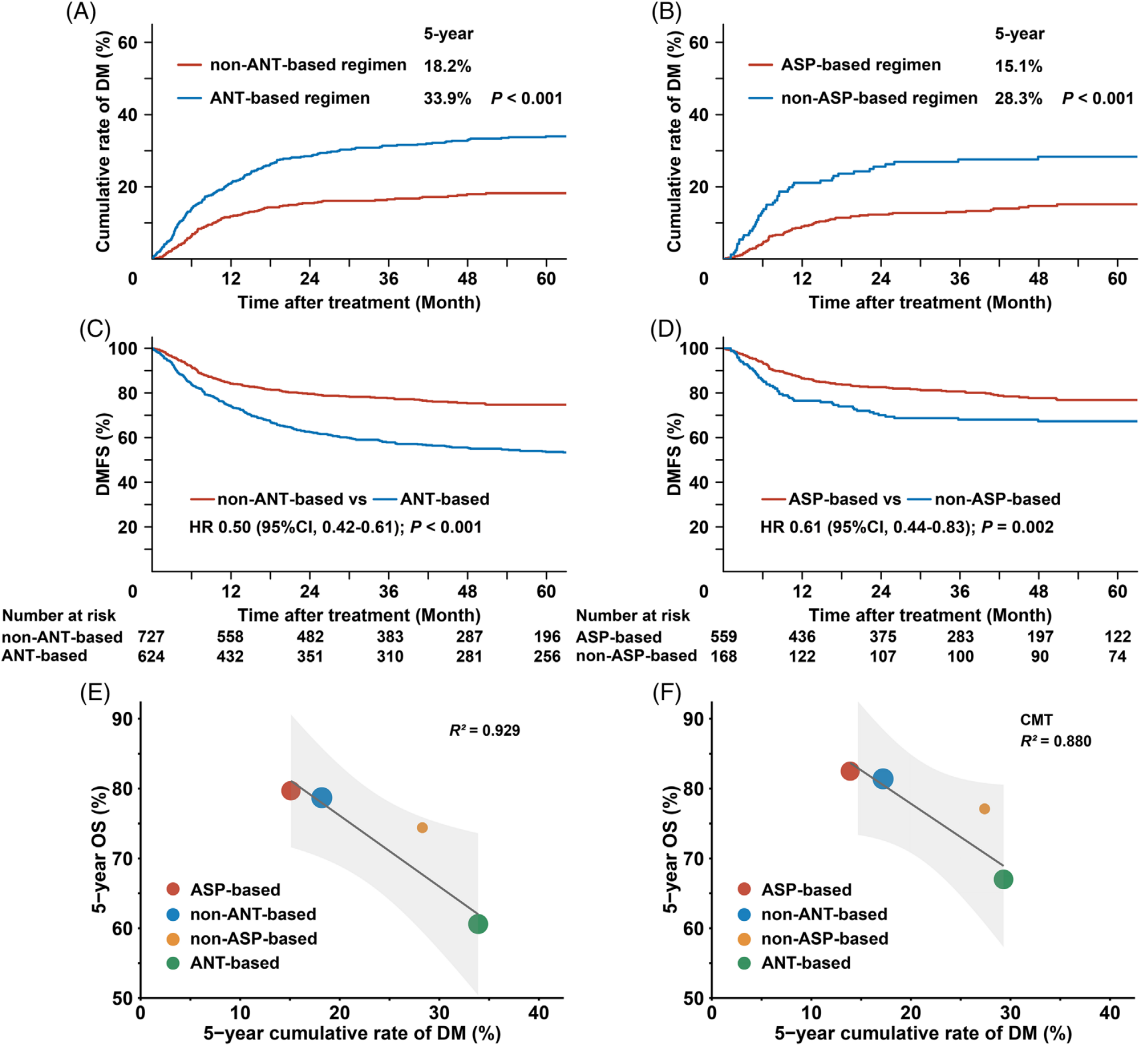
Association of contemporary chemotherapy regimens with DM rate, DMFS, and OS. Gray's cumulative incidence of DM between (A) non‐ANT‐based and ANT‐based regimens (*p* < 0.001), and (B) ASP‐based and non‐ASP‐based regimens (*p* < 0.001); and the comparison of DMFS between (C) non‐ANT‐based and ANT‐based regimens (*p* < 0.001), and (D) ASP‐based and non‐ASP‐based regimens (*p* = 0.002) in patients receiving chemotherapy with or without RT. Survival‐dependent linear regression analyses based on chemotherapy regimens revealed strong correlations (E) between 5‐year DM rates and 5‐year OS following chemotherapy with or without RT (*R* ^2^ = 0.929); and (F) between 5‐year DM rates and 5‐year OS following CMT (*R* ^2^ = 0.880). DM, distant metastasis; DMFS, distant metastasis‐free survival; OS, overall survival; ANT, anthracycline; ASP, asparaginase; RT, radiotherapy; CMT, combined modality therapy; *R* ^2^, determination coefficient; HR, hazard ratio; CI, confidence interval

Primary treatment with ASP‐based and non‐ANT‐based regimens was observed to associate with lower DM rates and better DMFS. The 5‐year cumulative rates of DM for non‐ANT‐based and ANT‐based regimens were 18.2% and 33.9%, respectively (*p* < 0.001; Figure 5A), while those of ASP‐based and non‐ASP‐based regimens were 15.1% and 28.3%, respectively (*P* < 0.001; Figure 5B).

For chemotherapy with or without RT, the 5‐year DMFS of non‐ANT‐based regimens was significantly higher than that of ANT‐based regimens (74.8% vs. 53.6%; HR 0.50; 95% CI 0.42–0.61; *p* < 0.001; Figure 5C). The IPTW‐adjusted 5‐year DMFS was 74.2% and 54.0% for non‐ANT‐based and ANT‐based regimens, respectively (HR 0.52; 95% CI 0.43–0.63; *p* < 0.001; Supplemental Figure 2A). In the setting of CMT, non‐ANT‐based regimens also showed significantly higher 5‐year DMFS than ANT‐based regimens (77.7% vs. 60.7%; HR, 0.53; 95% CI 0.43–0.67; *p* < 0.001). The IPTW‐adjusted 5‐year DMFS was 77.5% and 60.1% for non‐ANT‐based and ANT‐based regimens, respectively (HR 0.53; 95% CI 0.42–0.67; *p* < 0.001; Supplemental Figure 2C).

For non‐ANT‐based chemotherapy with or without RT, the 5‐year DMFS for ASP‐based regimens was significantly higher than that of non‐ASP‐based regimens (76.8% vs. 67.3%; HR 0.61; 95% CI 0.44–0.83; *p* = 0.002; Figure 5D). The IPTW‐adjusted 5‐year DMFS was 76.6% and 68.4% for ASP‐based and non‐ASP‐based regimens, respectively (HR 0.64; 95% CI 0.45–0.91; *p* = 0.012; Supplemental Figure 2B). For patients who received CMT, the 5‐year DMFS was also significantly higher with ASP‐based regimes (80.2% vs. 69.8%; HR 0.57; 95% CI 0.40–0.82; *p* = 0.002). The IPTW‐adjusted 5‐year DMFS for ASP‐based and non‐ASP‐based regimens was 80.2% and 69.6%, respectively (HR 0.57; 95% CI 0.38–0.85; *p* = 0.004; Figure S2D). Similar DMFS benefits were observed between non‐ANT‐based and ANT‐based regimens, and between ASP‐based regimen and non‐ASP‐based regimens for intermediate‐ and high‐risk early‐stage patients (Figure S3A–D). These results indicated that non‐ANT‐based and ASP‐based chemotherapy regimens, regardless of RT, played an important role in DMFS improvement.

### 3.8 Correlation between cumulative DM rates and OS after contemporary chemotherapy

Given the demonstrated OS benefits of contemporary chemotherapy in previous studies [8, 10, 18], the association of cumulative DM rates with OS based on primary chemotherapy regimens was assessed. With or without RT, the 5‐year OS was 78.7% for non‐ANT‐based regimens, 60.6% for ANT‐based regimens, 79.7% for ASP‐based regimens, and 74.4% for non‐ASP‐based regimens. Linear regression analysis revealed a strong correlation between 5‐year cumulative DM rates and 5‐year OS (determination coefficient, *R* ^2^ = 0.929; Figure 5E).

In the setting of CMT, the 5‐year OS was 81.4% for non‐ANT‐based regimens, 67.0% for ANT‐based regimens, 82.5% for ASP‐based regimens, and 77.1% for non‐ASP‐based regimens. The 5‐year cumulative DM rates were 17.2% for non‐ANT‐based regimens, 29.3% for ANT‐based regimens, 13.9% for ASP‐based regimens, and 27.4% for non‐ASP‐based regimens. Similarly, a strong correlation between 5‐year cumulative DM rates and 5‐year OS was observed (*R* ^2^ = 0.880; Figure 5F).

These results indicated that decreased DM rates following novel chemotherapy regimens were associated with increased OS probability.

## 4 DISCUSSION

The treatment landscape of early‐stage ENKTCL continues to evolve with novel risk‐adapted therapeutic strategies, optimized RT techniques, and modern chemotherapy regimens. However, the characteristics and patterns of DM, along with the associated outcomes, have not been fully elucidated. In this CLCG study, we identified the site‐specific patterns of DM following primary treatment of early‐stage ENKTCL, and further classified DM into four distinct subgroups. SST dissemination was observed to be most frequent and was less likely to occur with concurrent visceral organ or LAHS dissemination. The proposed classification of DM was found to be an independent prognostic factor for OS. A relatively favorable prognosis was shown in patients with SST or distant LN metastasis, solitary metastasis, and late‐onset DM. Contemporary chemotherapy regimens significantly decreased DM rates and improved DMFS and produced a linear relationship between the cumulative DM rates and OS. These findings have important clinical implications for treatment decision‐making and metastatic surveillance, as well as for the design of future prospective studies on this disease.

Based on the characteristics and correlations of DM sites in early‐stage ENKTCL after primary treatment, four subgroups with distinct prognoses were identified—distant LN, SST, extracutaneous sites, and LAHS. The survival of patients with SST involvement was significantly longer than those with extracutaneous site involvement or LAHS but was comparable to those with distant LN involvement. The heterogeneous clinical courses of these subgroups paralleled the variation in the time to DM and the number of DM sites. SST dissemination was the primary DM course of early‐stage ENKTCL. Similar to our previous finding, most patients (60.0%) presented with SST involvement without extracutaneous metastasis [26]. The observed propensities for cutaneous dissemination and localized SST lesions indicate that ENKTCL primarily involves a generalized homing phenomenon of NK/T‐cell lymphocytes to the skin [32]. This is in contrast to DLBCL, whereby LN, BM, and visceral organ dissemination, and thus the homing phenomenon of B‐cell lymphocytes to such organs, is more frequently observed [33]. Moreover, we found a very low frequency of CNS involvement, which does not justify the current use of CNS prophylaxis [34], and challenges the indications for methotrexate‐containing regimens as first‐line management of early‐stage ENKTCL.[12] LAHS represents a catastrophic event in ENKTCL, with a median survival time of only 1–4 months [28, 29]. Such distinctions in the course of DM may imply an underlying difference in biological behavior, which may suggest the need for distinct salvage treatment approaches. Patients in each category of their classification of DM sites may require different possible treatments to individualize salvage approach and develop strategies with the incorporation of DM‐site modifying therapies. Patients with CNS involvement need CNS‐active regimens, and those with localized SST involvement can be treated with chemoradiotherapy. Patients with intestinal lesions may require surgery. Further studies on the inherent mechanisms and clinical relevance of DM sites are warranted to guide the development of targeted therapy and immunotherapy.

The survival of patients with DM in this series was poor, with a median OS of 16.8 months from an initial treatment and a mere 4.3 months from DM onset. Most patients with DM deteriorated rapidly and died within 2 years, indicating the lack of effective salvage systemic treatment. In our previous CLCG studies [19–21, 35], the increase in survival probability and the decrease in annual failure hazard rates observed in RT‐treated early‐stage patients occurred in a risk‐dependent manner [35]. Patients who were progression‐free within 24 months after primary treatment demonstrated favorable long‐term outcomes, with OS being indistinguishable from those expected of age‐, sex‐, and country‐matched general populations [20]. Conversely, poor prognosis has been seen in patients with disease progression within 2 years (median subsequent OS, ∼6 months) [20, 21], and in those with uncured diseases (median OS, 13.2 months) [19]. Despite the extremely poor prognoses observed in patients with DM, a subgroup of our patients with solitary DM demonstrated a 3‐year subsequent OS of 62.9%, suggesting a potential for long‐term survival.

The concept of oligometastasis has been widely applied to solid tumors [36, 37], and represents the interim state between locally confined lesions and systemically disseminated disease. This, however, has not been used in lymphomas. In our study, the relatively favorable prognoses seen in patients with solitary metastasis, regardless of site, resembled the oligometastatic state, whereby metastatic capacity remains low over a prolonged period of time. The favorable prognosis of patients with SST metastasis may be partly due to the tendency for solitary metastasis in ENKTCL. No survival differences were found between SST and extracutaneous site involvement in the context of oligometastatic disease. In contrast, patients with multiple DM sites were associated with a worse prognosis and represented a more aggressive phenotype. The oligometastatic state provides a potential therapeutic window to halt the early‐phase progression of the disease. Previous studies have shown the success of RT in achieving long‐term survival among patients with localized cutaneous or locoregional metastasis [25, 26]. Therefore, integrating locoregional RT into second‐line systemic treatment interventions may provide survival benefits to patients with limited DM, even though systemic treatments are often expected for disseminated diseases. To enable the development of individualized treatment strategies, further studies are needed to elucidate the underlying pathophysiologies of solitary and multiple metastatic diseases.

Our study represents a critical step toward understanding the effects of modern chemotherapy regimens on the clinical outcomes of early‐stage ENKTCL. While the efficacy of contemporary chemotherapy in improving the OS of ENKTCL is well‐established [8, 10, 18], the relationship between DM rates and OS remains unclear, particularly in the context of primary RT for early‐stage patients. This large‐scale study confirmed that decreased cumulative DM rates by novel chemotherapy regimens were associated with increased OS probabilities. In previous CLCG studies [13, 18], we have demonstrated that good locoregional control with RT is associated with prolonged PFS and OS in early‐stage ENKTCL [13]. However, combining primary RT with ASP‐based regimen chemotherapy has shown limited effects on locoregional control [18], implying that contemporary chemotherapy improves survival by targeting distant occult micrometastatic diseases rather than via locoregional effects. Early‐stage ENKTCL can be considered both a systemic and localized disease. In the era of modern treatment, combined RT and chemotherapy have played an important role in eradicating both locoregional and metastatic diseases in early‐stage ENKTCL [16, 18]. In contrast to chemosensitive DLBCL, both locoregional and distant control should remain the main points of focus for the optimization of first‐line therapy and the eventual improvement of long‐term survival in early‐stage ENKTCL patients.

There are strengths and limitations. To the best of our knowledge, our study is the first to classify DM into four distinct subgroups based on clinical behavior, and to demonstrate a clear association between decreased DM rates and OS benefit with contemporary chemotherapy for early‐stage ENKTCL. We further found that the therapeutic efficacy of such chemotherapy regimens mostly relies on decreasing DM. However, several limitations need to be considered. First, the pathological and radiological data were not centrally reviewed, as DM was diagnosed independently in each institution. Second, the impact of salvage treatment on survival after disease progression was not investigated, as it is beyond the scope of this study. Originally, the CLCG dataset was designed to collect only baseline clinicopathological characteristics, initial treatments, and events during follow‐up. Third, our study cohort was limited to early‐stage ENKTCL patients who developed DM, which may render our results inapplicable to advanced‐stage patients primarily treated with ASP‐based chemotherapy, given the differences in first‐line treatments indicated between such stages of ENKTCL. It is emphasized that the results in this study cannot extrapolate to stage III/IV ENKTCL. Further work is needed to validate our findings in patients with advanced‐stage ENKTCL, and to enable the optimization of salvage treatment strategies for all stages of ENKTCL.

In summary, DM can be classified into four distinct subgroups of different prognoses. Contemporary chemotherapy is associated with decreased DM rates and improved DMFS. Our findings serve as an important benchmark for future therapeutic decisions, metastatic surveillance, and translational studies in the field of ENKTCL.

## AUTHOR CONTRIBUTIONS

YXL and SNQ designed the research; YXL, SNQ, XZ, XL, and BLQ collected and analyzed the data; XZ, XL, BLQ, SNQ, and YXL wrote the manuscript; all authors provided patient data, and approved the final manuscript.

## CONFLICT OF INTEREST

The authors declare that they have no conflict of interest.

## ETHICS STATEMENT

This study was approved by the Ethics Committee of Cancer Institute and Hospital, Chinese Academy of Medical Sciences (Approval No. 16‐043/1122).

## Supporting information

Supporting Information
