# Supplementary material for: Characteristics and prognosis of distant metastasis after primary treatment for early‐stage extranodal nasal‐type natural killer/T‐cell lymphoma from the China Lymphoma Collaborative Group database
Source: EJHaem. 2022 Nov 25;4(1):78–89. doi: 10.1002/jha2.613 (PMC9928645; doi:10.1002/jha2.613)
Supplement: Supplementary file 1 — Supporting Information [file JHA2-4-78-s001.docx]

**Supplemental Table 1.** Multivariable analysis of DMFS for early-stage ENKTCL patients.

**DMFS**

| **Variables** | **HR (95% CI)** | ***P*** |
| --- | --- | --- |
| Gender (female vs. male) | 0.93 (0.76-1.12) | 0.442 |
| Age (>60 vs. ≤60) | 1.59 (1.28-1.98) | <0.001 |
| B symptoms (yes vs. no) | 0.99 (0.83-1.18) | 0.891 |
| ECOG score (≥2 vs. 0–1) | 2.30 (1.70-3.10) | <0.001 |
| Ann Arbor stage (II vs. I) | 1.27 (1.06-1.53) | 0.010 |
| Elevated LDH (yes vs. no) | 1.51 (1.26-1.81) | <0.001 |
| PTI (yes vs. no) | 1.62 (1.35-1.95) | <0.001 |
| Primary site |  | <0.001 |
| Extranasal-UADT vs nasal | 1.26 (1.03-1.54) | 0.022 |
| Extra-UADT vs nasal | 2.21 (1.52-3.21) | <0.001 |

Abbreviations: DMFS, distant metastasis-free survival; ENKTCL, extranodal nasal-type natural killer/T-cell lymphoma; HR, hazard ratio; CI, confidence interval; ECOG, Eastern Cooperative Oncology Group; LDH, lactate dehydrogenase; PTI, primary tumor invasion; UADT, upper-aerodigestive tract.

# Supplemental Figure 1

**Stage I-II patients from 6 institutions (n = 1619)**

**Patients with information on DM in 6 institutions (n = 1903)**

**Excluded：**

- **Stage III-IV patients (n = 284)**

**ENKTCL patients treated in 20 institutions between 2000 and 2018 in the CLCG database**

**(n = 3306)**

**Excluded：**

- **Patients from 14 institutions without information on DM (n = 1403)**

**Supplemental Figure 2**


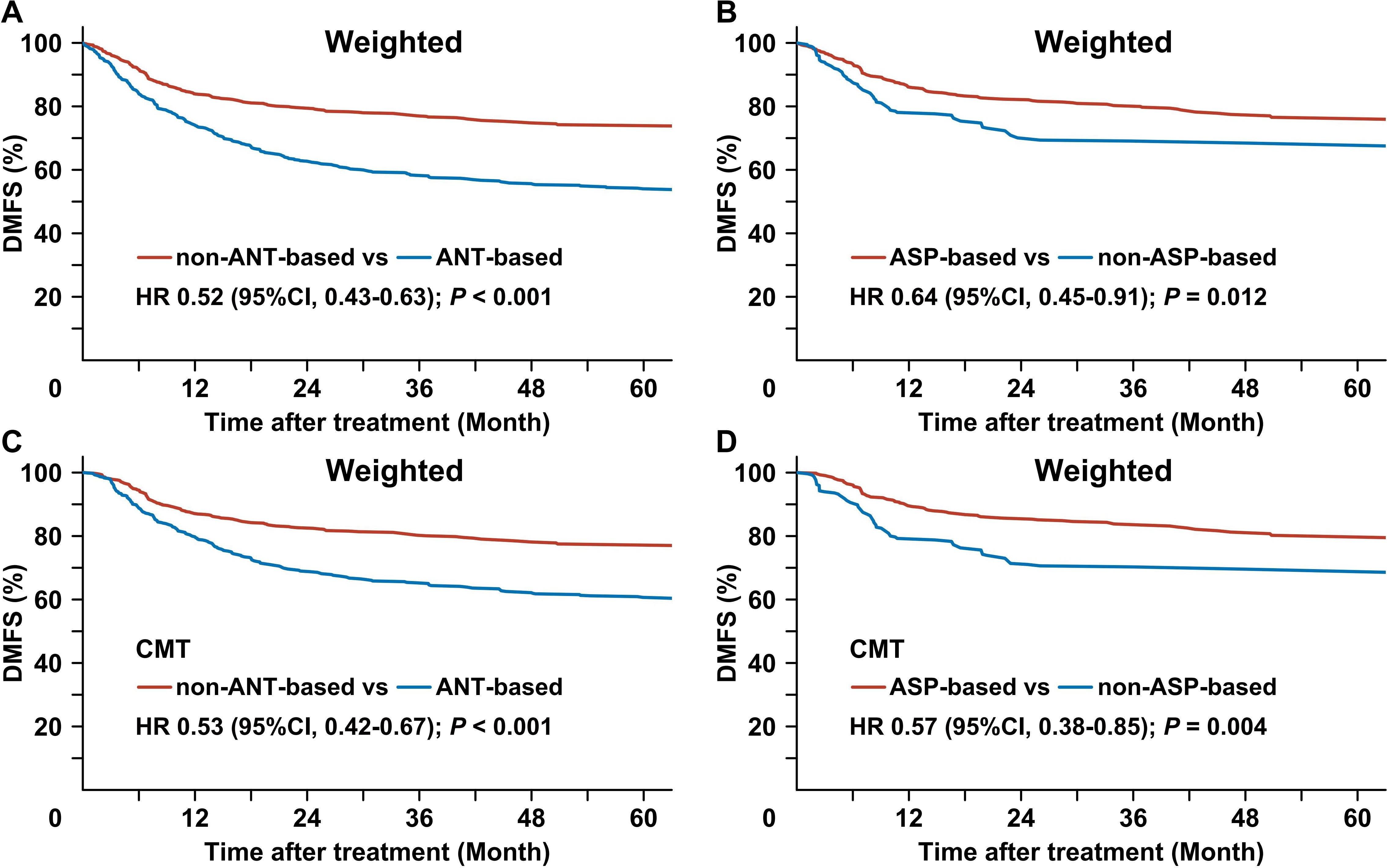


**Supplemental Figure 3**


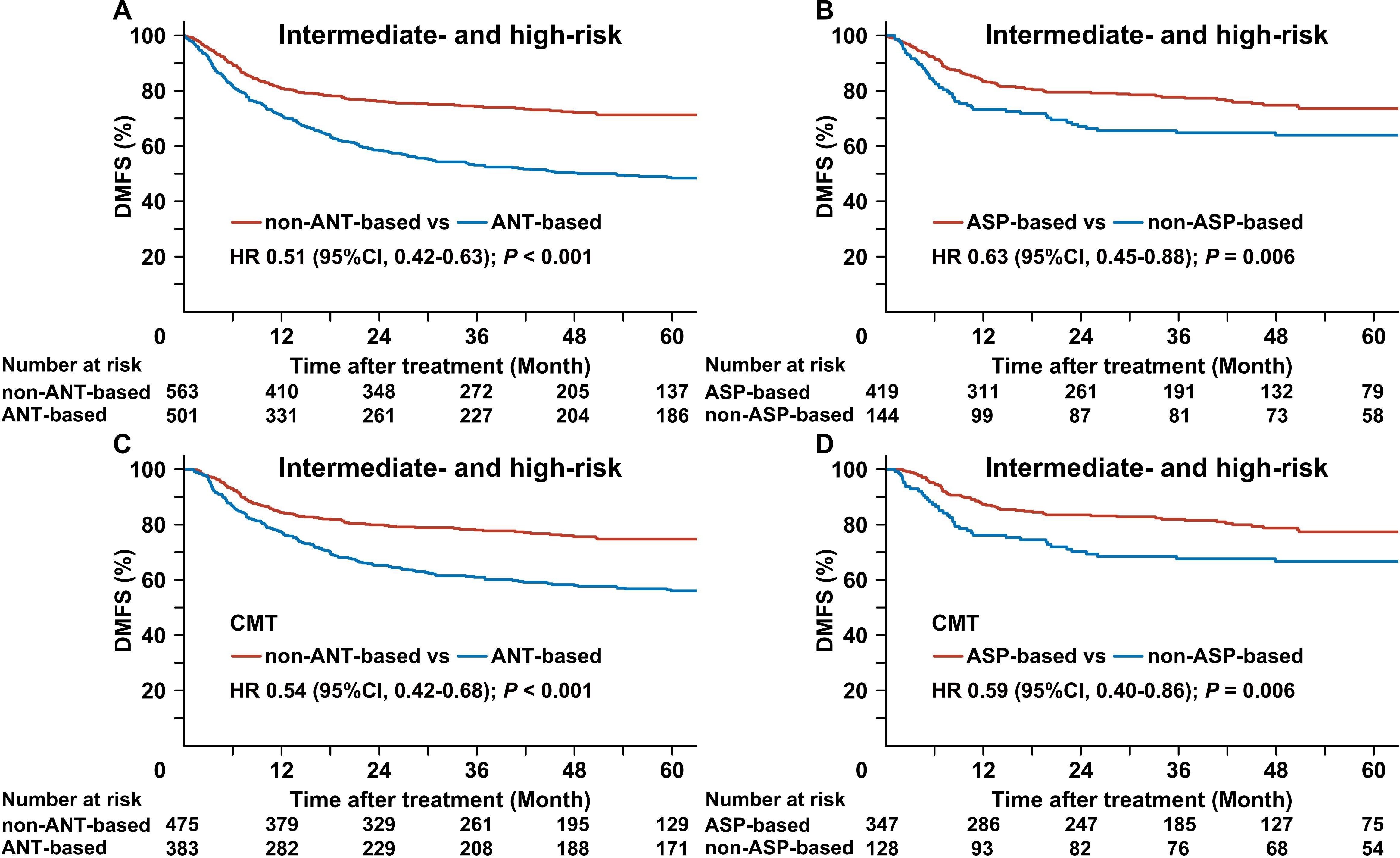


**SUPPLEMENTAL FIGURE LEGENDS**

**Supplemental Figure 1. CONSORT diagram.** ENKTCL, extranodal nasal-type natural killer/T-cell lymphoma; CLCG, China Lymphoma Collaborative Group; DM, distant metastasis.

**Supplemental Figure 2. Effect of contemporary chemotherapy regimens on DMFS after IPTW adjustment.** The comparison of DMFS between (**A**) non-ANT- based and ANT-based regimens (*P*<0.001), and (**B**) ASP-based and non-ASP-based regimens (*P*=0.012) in patients receiving chemotherapy with or without RT; and the comparison of DMFS between (**C**) non-ANT-based and ANT-based regimens (*P*<0.001), and (**D**) ASP-based and non-ASP-based regimens (*P*=0.004) in patients receiving CMT. DMFS, distant metastasis-free survival; ANT, anthracycline; ASP, asparaginase; RT, radiotherapy; CMT, combined modality therapy; HR, hazard ratio; CI, confidence interval.

**Supplemental Figure 3. Effect of contemporary chemotherapy regimens on DMFS for intermediate- and high-risk early-stage patients.** The comparison of DMFS between (**A**) non-ANT-based and ANT-based regimens (*P*<0.001), and (**B**) ASP-based and non-ASP-based regimens (*P*=0.006) in patients receiving chemotherapy with or without RT; and the comparison of DMFS between (**C**) non- ANT-based and ANT-based regimens (*P*<0.001), and (**D**) ASP-based and non-ASP- based regimens (*P*=0.006) in patients receiving CMT. DMFS, distant metastasis-free

survival; ANT, anthracycline; ASP, asparaginase; RT, radiotherapy; CMT, combined modality therapy; HR, hazard ratio; CI, confidence interval.
